# Extensive Hair-Shaft Elongation by Isolated Mouse Whisker Follicles in Very Long-Term Gelfoam® Histoculture

**DOI:** 10.1371/journal.pone.0138005

**Published:** 2015-09-30

**Authors:** Wenluo Cao, Lingna Li, Sumiyuki Mii, Yasuyuki Amoh, Fang Liu, Robert M. Hoffman

**Affiliations:** 1 AntiCancer Inc., San Diego, CA, United States of America; 2 Department of Surgery, University of California San Diego, San Diego, CA, United States of America; 3 Department of Anatomy, Second Military Medical University, Shanghai, China; 4 Department of Dermatology, Kitasato University, Sagimahara, Japan; National Cancer Institute, UNITED STATES

## Abstract

We have previously studied mouse whisker follicles in Gelfoam® histoculture to determine the role of nestin-expressing plutipotent stem cells, located within the follicle, in the growth of the follicular sensory nerve. Long-term Gelfoam® whisker histoculture enabled hair follicle nestin-expressing stem cells to promote the extensive elongation of the whisker sensory nerve, which contained axon fibers. Transgenic mice in which the nestin promoter drives green fluorescent protein (ND-GFP) were used as the source of the whiskers allowing imaging of the nestin-expressing stem cells as they formed the follicular sensory nerve. In the present report, we show that Gelfoam®-histocultured whisker follicles produced growing pigmented and unpigmented hair shafts. Hair-shaft length increased rapidly by day-4 and continued growing until at least day-12 after which the hair-shaft length was constant. By day-63 in histoculture, the number of ND-GFP hair follicle stem cells increased significantly and the follicles were intact. The present study shows that Gelfoam® histoculture can support extensive hair-shaft growth as well as hair follicle sensory-nerve growth from isolated hair follicles which were maintained over very long periods of time. Gelfoam® histoculture of hair follicles can provide a very long-term period for evaluating novel agents to promote hair growth.

## Introduction

We previously established the technique of long-term Gelfoam® histoculture of isolated whisker follicles from nestin-driven green fluorescent protein (ND-GFP) transgenic mice [1]. ND-GFP-expressing pluripotent hair follicle stem cells were discovered by our laboratory and observed to be located above the hair follicle bulge. The pluripotent hair follicle stem cells were able to differentiate into neurons, heart muscle cells [2] and other cell types in vitro [3,4]. The nestin-expressing hair follicle stem cells promoted the recovery of pre-existing axons when they were transplanted to the severed sciatic nerve [5] or injured spinal cord [6] of nude mice. We have previously demonstrated that the whisker hair follicle contains nestin-expressing stem cells in the dermal papilla (DP) as well as in the bulge area (BA), with their origin in the BA [7].

ND-GFP-expressing stem cell trafficking in real time between the BA and DP in Gelfoam® histoculture was monitored by confocal imaging to determine the fate of the stem cells. The stem cells trafficked from the BA toward the DP area and extensively grew out onto Gelfoam® forming nerve-like structures over a two-week period [1]. We subsequently observed that β-III tubulin-positive fibers, highly enriched in ND-GFP-expressing cells, extending up to 500 μm from the whisker nerve stump in Gelfoam® histoculture. The fibers had growth cones on their tips expressing F-actin indicating that the fibers growing from the whisker follicle sensory nerve stump were growing axons, which were highly enriched in ND-GFP cells that appeared to play a major role in axon fiber elongation [8].

We previously reported the long-term growth, shaft elongation, and spontaneous regression of human hair follicles in Gelfoam® histoculture of intact scalp skin. Human scalp skin, with abundant hair follicles in various stages of the hair growth cycle, was grown for up to 40 days on Gelfoam®. Isolated human scalp follicles placed on Gelfoam® also supported hair shaft elongation [9]. When mouse skin was histocultured on Gelfoam®, hair shafts elongation was observed for up to 14 days [10].

In the present report, we used Gelfoam® histoculture of isolated whiskers from ND-GFP mice to determine hair-shaft growth as well as proliferation of ND-GFP stem cells over very long periods. Our results demonstrate that extensive hair-shaft elongation occurred in Gelfoam® histoculture of mouse whisker follicles from ND-GFP transgenic mice. Gelfoam® histoculture of mouse whisker follicles thus supports both extensive hair-shaft growth as well as follicle sensory nerve elongation. The Gelfoam® histocultured follicles remained intact with brightly-fluorescent proliferating ND-GFP stem cells for at least 63 days.

## Materials and Methods

### Ethics statement

All animal studies were conducted with an AntiCancer, Inc., Institutional Animal Care and Use Committee (IACUC)-protocol specifically approved for this study and in accordance with the principles and procedures outlined in the National Institute of Health Guide for the Care and Use of Animals under Assurance Number A3873-1. In order to minimize any suffering of the animals, anesthesia and analgesics were used for all surgical experiments. Animals were anesthetized by intramuscular injection of a 0.02 ml solution of 20 mg/kg ketamine, 15.2 mg/kg xylazine, and 0.48 mg/kg acepromazine maleate. The response of animals during surgery was monitored to ensure adequate depth of anesthesia. Ibuprofen (7.5 mg/kg orally in drinking water every 24 hours for 7 days post-surgery) was used in order to provide analgesia post-operatively in the surgically-treated animals. The animals were observed on a daily basis and humanely sacrificed by CO_2_ inhalation when they met the following humane endpoint criteria: prostration, skin lesions, significant body weight loss, difficulty breathing, epistaxis, rotational motion and body temperature drop. The animals were necessary as a source of nestin-GFP-expressing hair follicles. The endpoint was the measurement of the hair shaft elongation during the in vitro histoculture of the whisker hair follicles. Animals were housed with no more than 5 per cage. Animals were housed in a barrier facility on a high efficiency particulate air (HEPA)-filtered rack under standard conditions of 12-hour light/dark cycles. The animals were fed an autoclaved laboratory rodent diet (S1 ARRIVE Checklist).

### Mice

Transgenic mice with nestin-driven GFP (ND-GFP) (4 to 8 weeks) (AntiCancer, Inc., San Diego, CA) were used as a source of whisker follicles. All animal studies were conducted in accordance with the principals and procedures outlined in the National Institute of Health Guide for the Care and Use of Animals under Assurance Number A3873-1 [8].

### Isolation of vibrissa-follicles (Whiskers)

The whisker pad was dissected to obtain isolated vibrissae follicles with forceps and fine needles, from mice anesthetized using a ketamine mixture (intramuscular injection of a 0.02 ml solution of 20 mg/kg ketamine, 15.2 mg/kg xylazine, and 0.48 mg/kg acepromazine maleate), using a binocular microscope (MZ6, Leica, Weztlar, Germany). The isolated vibrissae hair follicles with their capsules were used for Gelfoam® histoculture [8].

### Gelfoam® histoculture and growth medium

The vibrissae hair follicles were placed in DMEM-F12 (GIBCO/BRL, Grand Island, NY) containing B-27 (2.5%) (GIBCO/BRL), N2 (1%) (GIBCO/BRL), and 1% penicillin and streptomycin (GIBCO/BRL) on sterile Gelfoam® (Pharmacia and Upjohn Co., Kalamazoo, MI) hydrated in the medium. The Gelfoam® histocultured follicles were incubated at 37°C, 5% CO_2_ 100% humidity. The medium was changed every other day [1, 8].

### Confocal laser scanning microscopy

A confocal laser scanning microscope (FV1000, Olympus Corp., Tokyo, Japan) was used for two- (X,Y) and three-dimensional (3D, X,Y,Z) high-resolution imaging of the vibrissa follicles in Gelfoam® histoculture. Fluorescence images were obtained using the 4×/0.10 Plan N, 10×/0.30 Plan-NEOFLUAR, 20×/0.50 UPlan FL N, and 20×/1.00w XLUMplan FL objectives [11].

### Statistical analyses

Differences were obtained using the unpaired Student’s *t*-test. The significance level is p ≤ 0.05.

## Results and Discussion

### Nestin-GFP hair-shaft elongation in Gelfoam^®^ histoculture

**Fig 1:**
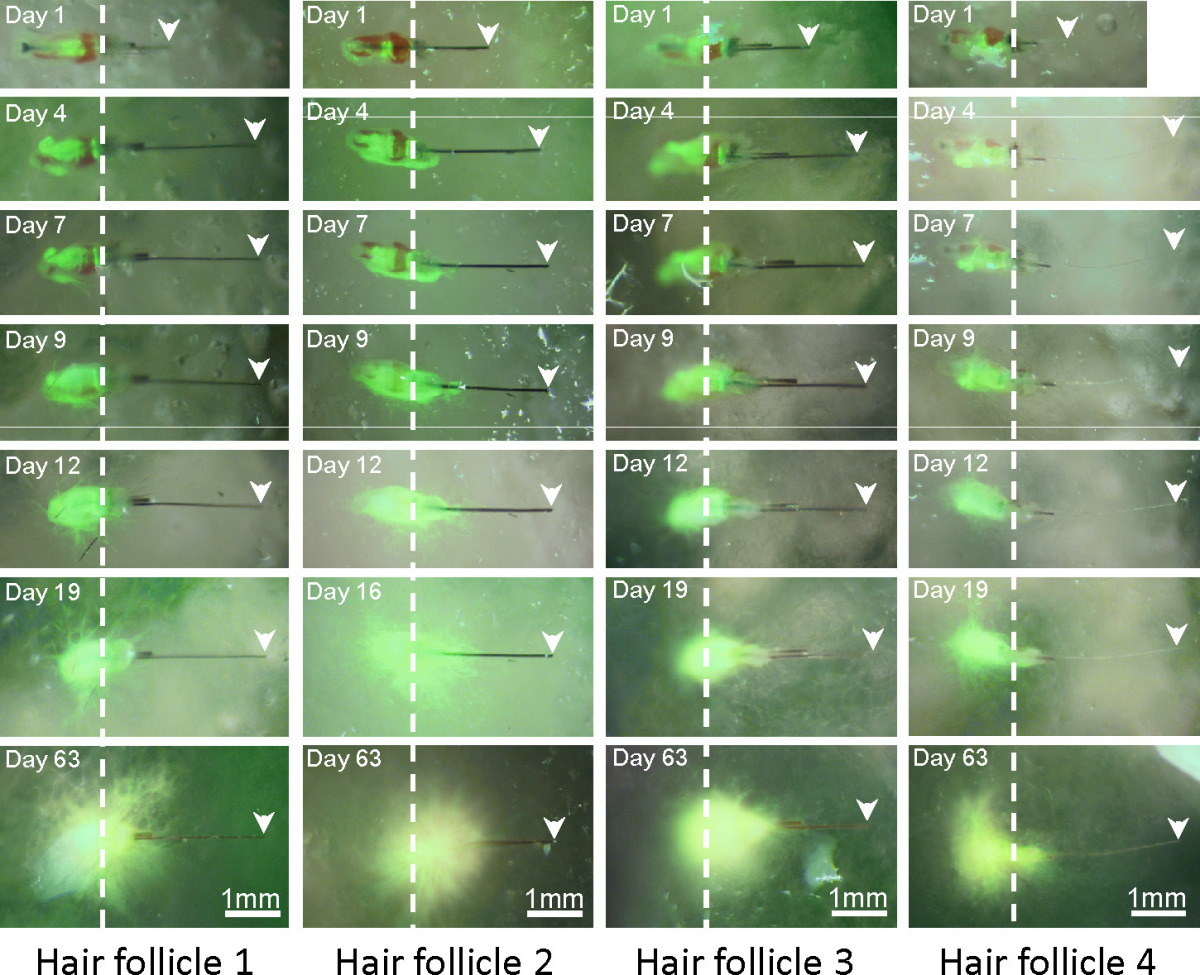
Hair shaft elongation of mouse whiskers in Gelfoam^®^ histoculture. Time-course images of hair shaft growth from individual mouse whisker follicles, isolated from nestin-driven green fluorescent protein (ND-GFP) mice, histocultured on Gelfoam®. Green fluorescence was from the ND-GFP-expressing stem cells in the whisker hair follicles which were enriched during 63 days of histoculture *in vitro*. Hair shafts lengthened rapidly in the first 4 days, extended over 9–12 days, and remained the same length until day 63.

**Fig 2:**
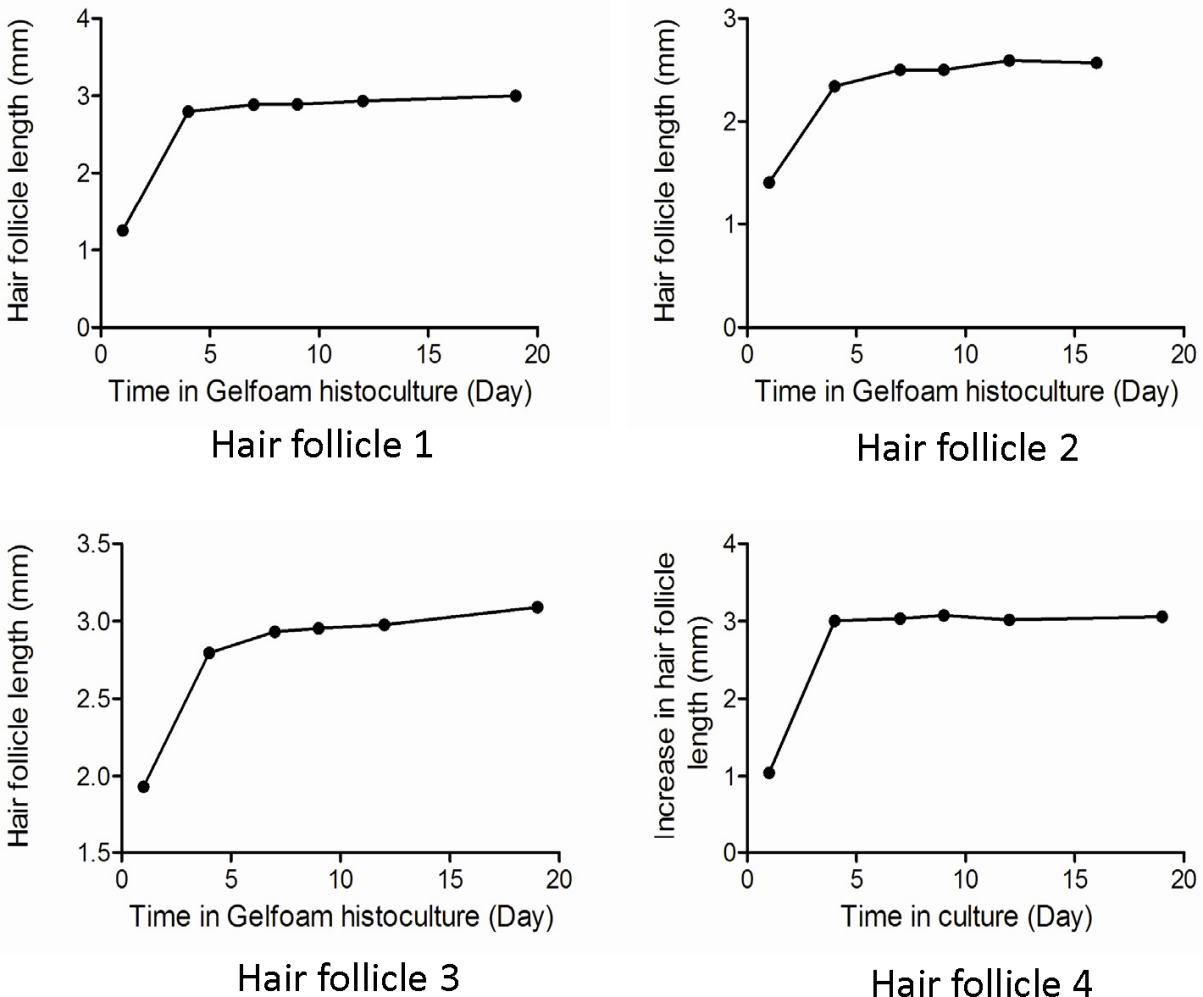
Graphs quantifying the increase of shaft length over time in individual follicles during Gelfoam® histoculture.

**Fig 3:**
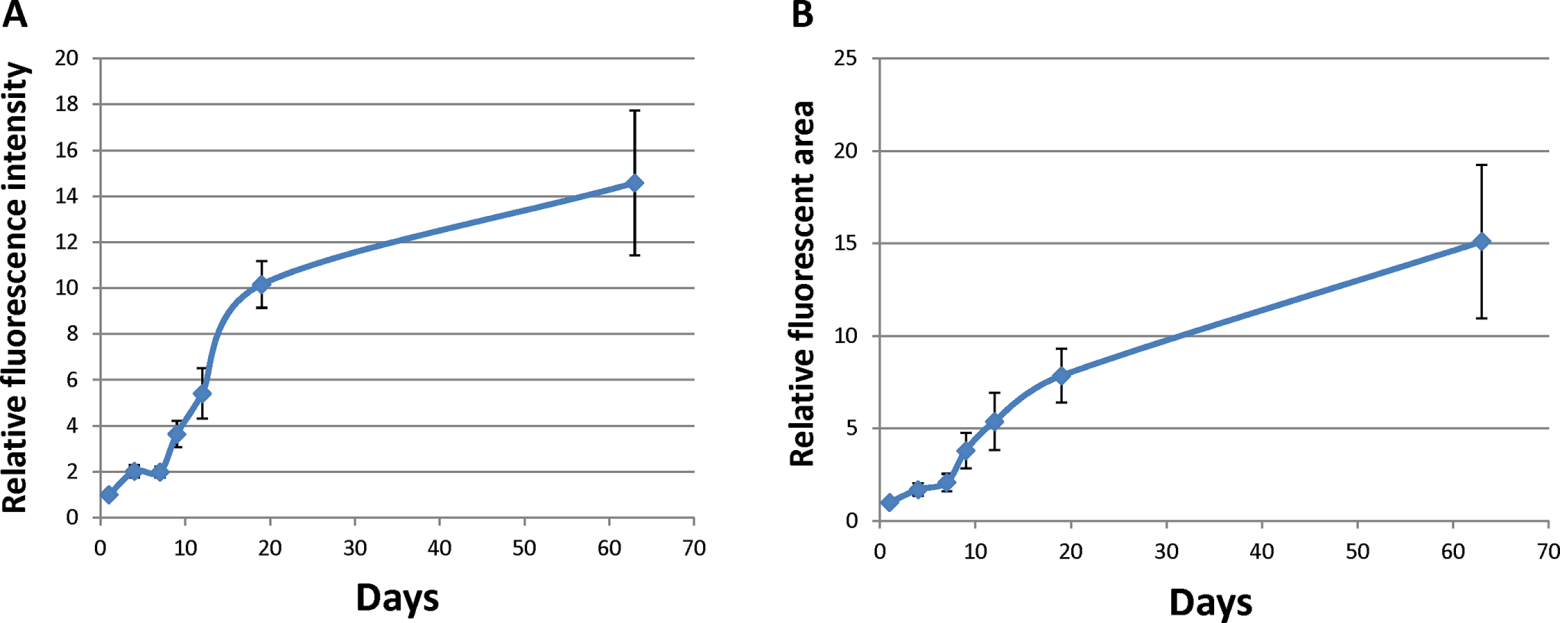
Graph quantifying the time-course increase of hair follicle stem cell GFP fluorescence intensity (A) and fluorescent area (B). *p*<0.01 in increase of fluorescent area and fluorescence intensity at day 63 compared to day 1.

When whisker hair follicles were freshly isolated, the follicle was covered by a rigid and intact capsule filled with red blood (Fig 1). The isolated follicles with the capsule were then placed on Gelfoam® histoculture. Hair-shaft length in the follicles increased by 1.32 ± 0.27 mm by day 4 compared to day 1 and kept growing at day 7 (1.42 ± 0.24 mm) and day 9 (1.46 ± 0.24 mm) compared to day 1. By day-12, the hair shaft length was 1.50 ± 0.22 mm (p<0.001 compared to day-1) and remained constant until day 63 (Figs 1 and 2). At day-63 of hair follicle histoculture, the ND-GFP-expressing stem cell follicles had a large increase in relative fluorescence intensity and fluorescent area (*p* < 0.001 for both). The large increase in ND-GFP expression of the stem cells indicates their extensive proliferation and activity, as well as the very long-term viability of the follicles in Gelfoam® histoculture. Thus ND-GFP-expressing stem cells increased over the 63-day histoculture period even though hair shaft elongation appeared to cease by day 20 (Figs 2 and 3).

We previously reported on hair-shaft elongation from human scalp and mouse skin in Gelfoam® histoculture [9, 10]. However, in the Gelfoam® histoculture of human scalp skin, hair-shaft growth was 0.86 ± 0.18 mm over 5 days [9] and 0.7–1.10 ± 0.22 mm over 5–14 days of the histocultured mouse skin [10]. Isolated human hair follicles in Gelfoam® histoculture grew only 0.49 ± 0.06 mm for 5 days [9].

The present report demonstrates improved hair shaft growth of isolated hair follicles on Gelfoam®. Isolated free-floating follicles were previously observed to produce elongating hair shafts but apparently were not viable for very long periods of time [12, 13]. Gelfoam® supports both hair shaft and nerve growth [8] of isolated whisker follicles and maintained viability of the follicles for at least 63 days, much longer than free-floating follicles, which can enable long-term experimentation. Thus, the present report demonstrates an improvement of hair follicle histoculture with increased hair-shaft growth, increased period of histoculture, as well as increasing stem cell fluorescence intensity and fluorescent area for at least 63 days. The strong increase in hair-follicle stem-cell ND-GFP fluorescence indicates increased activity and the proliferation of the stem cells. It is notable that the greatest increase in stem cell fluorescence is during the period of hair-shaft elongation (Figs 2 and 3).

Future experiments will explore the use of Gelfoam® hair follicle histoculture for enhancement of hair growth by experimental agents which could be tested over long periods of time which provides the opportunity to evaluate long-term efficacy and to determine on the rate and extent of hair-shaft elongation. Gelfoam® histoculture of whisker follicles is useful to study both hair shaft and follicle sensory nerve growth [8] over a long time period. Future experiments will also investigate the relationship of nestin-expressing stem cell activity and proliferation to hair-shaft elongation and why the elongation ceases before day 20.

## Dedication

This paper is dedicated to the memory of A. R. Moossa, M.D.

## Supporting Information

S1 ARRIVE Checklist ARRIVE checklist. (PDF)
